# A quantitative high resolution assessment of myocardial blood flow from contrast-enhanced first-pass magnetic resonance perfusion imaging: microsphere validation in a magnetic resonance compatible free beating explanted pig heart model

**DOI:** 10.1186/1532-429X-15-S1-E19

**Published:** 2013-01-30

**Authors:** Andreas Schuster, Matthew Sinclair, Niloufar Zarinabad, Masaki Ishida, Jeroen P van den Wijngaard, Matthias Paul, Pepijn van Horssen, Shazia T Hussain, Divaka Perera, Tobias Schaeffter, Jos A Spaan, Maria Siebes, Eike Nagel, Amedeo Chiribiri

**Affiliations:** 1 Division of Imaging Sciences and Biomedical Engineering, King's College London, London, UK; 2 Department of Cardiology and Pneumology and Heart Research Center, Georg-August-University, Göttingen, Germany; 3 Department of Biomedical Engineering & Physics, Academic Medical Centre, Amsterdam, Netherlands

## Background

The purpose of this study was to test the feasibility and validate quantification of myocardial perfusion using cardiovascular magnetic resonance (CMR) imaging with high-resolution voxel-wise assessment. An isolated perfused magnetic resonance (MR) compatible free beating pig heart model allows very accurate titration of myocardial blood flow (MBF). Regional MBF can be determined with an imaging cryomicrotome using fluorescently-labeled microspheres with excellent accuracy. We used this experimental set-up to assess the ability of CMR perfusion imaging to non-invasively quantify transmural distribution of MBF using a voxel-wise high-resolution assessment of state of the art CMR perfusion techniques.

## Methods

MR myocardial perfusion imaging was performed in explanted blood perfused free beating pig hearts at 1.5 Tesla (n=4) and 3 Tesla (n=4). Images were acquired during normal resting flow (100%), 50% flow and during adenosine induced hyperemia in control and coronary occlusion conditions. Fluorescently-labeled microspheres and known coronary blood flow were the reference standard for MBF validation. MBF quantification of the time-signal intensity curves was performed using a Fermi function approximation basis deconvolution.

## Results

High-resolution voxel-wise assessment of perfusion was able to distinguish between occluded and remote myocardium for all flow conditions (p<0.05). Furthermore perfusion at rest was distinguished from perfusion during 50% flow and hypaeremic perfusion at 1.5 Tesla (1.22±0.92 ml/min/g, 0.56±0.37 ml/min/g and 2.21±1.67 ml/min/g respectively, p<0.05) and at 3 Tesla (0.99±0.31 ml/min/g, 0.55±0.26 ml/min/g and 2.08±0.81 ml/min/g respectively, p<0.05).

CMR derived MBF estimates correlated well with the microspheres at the AHA segmental level both at 1.5 Tesla (r=0.94, p<0.001) and 3 Tesla (r=0.96, p<0.001). There was also a strong correlation, after subdivision of the segments (~2% left ventricular myocardium), at the subendocardial, mid-myocardial and subepicardial level at 1.5 Tesla (r=0.93, r=0.9, r=0.88, p<0.001 respectively) and at 3 Tesla (r=0.91, r=0.95, r=0.84, p<0.001 respectively).

## Conclusions

CMR derived high-resolution voxel-wise quantitative blood flow assessment is feasible and very accurate as compared to microspheres in regions of interest of around 2% left ventricular myocardium. This technique is suitable for both clinically used field strengths and may provide the tools to assess extent and severity of myocardial ischemia.

## Funding

Andreas Schuster is a British Heart Foundation (BHF) Clinical Research Fellow (FS/10/029/28253) and received grant support from the BHF (RE/08/003) and the Biomedical Research Centre (BRC-CTF 196). Matt Sinclair receives support from the Engineering and Physical Sciences Research Council (EP/H046410/1). Eike Nagel receives grant support from BHF (RE/08/003), the Wellcome Trust and Engineering and Physical Sciences Research Council (EPSRC, WT 088641/Z/09/Z) and the National Institute for Health Research (NIHR) via the comprehensive BRC award to Guy's and St Thomas' NHS Foundation Trust in partnership with King's College London and King's College Hospital NHS Foundation Trust. Amedeo Chiribiri was funded by the Wellcome Trust and EPSRC under grant number WT 088641/Z/09/Z.

Jeroen P. H. M. van den Wijngaard is funded by a VENI grant of the Netherlands Organization for Scientific Research (NWO/ZonMw 916.11.171). This study was further supported by grants to the AMC from the Netherlands Heart Foundation (NHS 2006B186 and 2006B226), the Netherlands Organization for Health Research and Development (ZonMw 91105008 and 91112030), and the European Community (FP7-2007-224495: euHeart project).

